# The Evolution of Quorum Sensing in Bacterial Biofilms

**DOI:** 10.1371/journal.pbio.0060014

**Published:** 2008-01-29

**Authors:** Carey D Nadell, Joao B Xavier, Simon A Levin, Kevin R Foster

**Affiliations:** 1 Department of Ecology and Evolutionary Biology, Princeton University, Princeton, New Jersey, United States of America; 2 Center for Systems Biology, Harvard University, Bauer Laboratory, Cambridge, Massachusetts, United States of America; University of Arizona, United States of America

## Abstract

Bacteria have fascinating and diverse social lives. They display coordinated group behaviors regulated by quorum-sensing systems that detect the density of other bacteria around them. A key example of such group behavior is biofilm formation, in which communities of cells attach to a surface and envelope themselves in secreted polymers. Curiously, after reaching high cell density, some bacterial species activate polymer secretion, whereas others terminate polymer secretion. Here, we investigate this striking variation in the first evolutionary model of quorum sensing in biofilms. We use detailed individual-based simulations to investigate evolutionary competitions between strains that differ in their polymer production and quorum-sensing phenotypes. The benefit of activating polymer secretion at high cell density is relatively straightforward: secretion starts upon biofilm formation, allowing strains to push their lineages into nutrient-rich areas and suffocate neighboring cells. But why use quorum sensing to *terminate* polymer secretion at high cell density? We find that deactivating polymer production in biofilms can yield an advantage by redirecting resources into growth, but that this advantage occurs only in a limited time window. We predict, therefore, that down-regulation of polymer secretion at high cell density will evolve when it can coincide with dispersal events, but it will be disfavored in long-lived (chronic) biofilms with sustained competition among strains. Our model suggests that the observed variation in quorum-sensing behavior can be linked to the differing requirements of bacteria in chronic versus acute biofilm infections. This is well illustrated by the case of Vibrio cholerae, which competes within biofilms by polymer secretion, terminates polymer secretion at high cell density, and induces an acute disease course that ends with mass dispersal from the host. More generally, this work shows that the balance of competition within and among biofilms can be pivotal in the evolution of quorum sensing.

## Author Summary

Bacteria are increasingly recognized as highly interactive organisms with complex social lives, which are critical to their capacity to cause disease. In particular, many species inhabit dense, surface-bound communities, termed *biofilms*, within which they communicate and respond to local cell density through a process known as *quorum sensing*. Enormous effort has been devoted to understanding the genetics and biochemistry of biofilm formation and quorum sensing, but how and why they evolve remain virtually unexplored. Many bacteria use quorum sensing to regulate the secretion of sticky extracellular slime, an integral feature of biofilm life. Intriguingly, however, some pathogenic species turn on slime production at high cell density, whereas others turn it off. Using an individual-based model of biofilm growth, we investigated why different species use quorum sensing to control slime production in opposite ways. The secret underlying this variation appears to reside in the nature of infections. Turning slime on at high cell density can allow one strain to suffocate another when competition is intense, as occurs in long-lived chronic infections. Meanwhile, turning slime secretion off at high cell density can benefit a strain causing an acute infection by allowing rapid growth before departing the host.

## Abstract 3

A novel exploration of intercellular communication in bacterial groups reveals why different species use quorum sensing to control slime secretion in opposite ways and suggests that these differences arise from evolutionary competition among strains.

## Introduction

Once perceived as organisms that rarely interact, bacteria are now known to lead highly social lives [1–3]. Central to this sociality is an ability to detect local cell density and thereby coordinate group behaviors [4–6]. This ability, termed *quorum sensing*, functions through the secretion and detection of autoinducer molecules, which accumulate in a cell density-dependent manner. When autoinducer concentrations reach a threshold level, quorum-sensing cells respond, allowing them to modulate behaviors whose efficacy and fitness benefits depend upon the presence, or absence, of other cells. Traits under quorum-sensing control include surface attachment [7], extracellular polymer production [8–10], biosurfactant synthesis [11], sporulation [12], competence [13], bioluminescence [14,15], and the secretion of nutrient-sequestering compounds and virulence factors [16–18]. Quorum sensing is also phylogenetically widespread, which suggests an early origin in bacterial evolution [19].

In addition to sensing and responding to the presence of other cells, many bacteria form multicellular surface-bound aggregates, or biofilms, whose remarkable feats of persistence are the scourge of both medicine and industry [5,6,20–24]. Accordingly, biofilms confer on their members considerable advantages, including the ability to resist challenges from predators, antibiotics, and host immune systems [6,20,25–27]. Quorum sensing and biofilm formation are often closely linked, and it is likely that their interaction is central to the pathogenesis of many bacterial infections [8–10,28–30]. The effects of quorum sensing, however, are highly variable and depend upon both the species under observation and the experimental conditions [28]. Four studies have emphasized how the potential for competition and conflict among strains of bacteria can shape the evolution of quorum sensing [31–34], but none have addressed biofilm formation. An open challenge for microbiology, therefore, is to disentangle the ecological and evolutionary processes that drive quorum sensing and biofilm phenotypes and, in particular, their interaction.

A defining feature of many biofilm-forming bacteria is the secretion of extracellular polymeric substances (EPS). These polymers, which consist largely of polysaccharide and smaller amounts of protein and DNA, form a matrix in which bacterial cells are embedded [5,6]. Recent empirical and theoretical work has shown that by secreting EPS, individual bacteria can both help and harm cells in their neighborhood and strongly affect the evolutionary dynamics within biofilms [35–38]. Using an individual-based biofilm simulation framework, Xavier and Foster [36] demonstrated that EPS production can provide an advantage to secreting strains by allowing them to push their descendent cells up into areas of high nutrient availability while suffocating any neighboring cells that do not produce EPS.

EPS secretion is under quorum-sensing control in a number of bacterial model systems. Many species, including the pathogen Pseudomonas aeruginosa, activate EPS production at high cell density [8,10]. The evolutionary rationale for this strategy seems clear: it increases the likelihood that polymer secretion will only occur in the biofilm state, where it affords a competitive advantage, and not in the planktonic state, where it is presumably a waste of resources [36]. Unexpectedly, other species behave quite differently. The human enteric pathogen Vibrio cholerae initiates EPS secretion after attaching to a surface and losing flagellar activity [39,40]. Subsequently, in a manner opposite to P. aeruginosa, V. cholerae halts EPS secretion once it reaches its high cell density quorum-sensing threshold [9,39]. Here, we explore evolutionary explanations for this variability in quorum-sensing control of EPS production using an individual-based model of biofilm formation [36]. In particular, we ask why do some species activate the biofilm-specific trait of polymer secretion at high cell density, while others terminate polymer secretion at high cell density?

## Methods/Results

We follow pairwise evolutionary competitions between strains that differ both in their ability to produce extracellular polymeric substances (EPS) and the extent to which this behavior is under quorum-sensing control. For our simulation study, we focus on three strains with the following behavior: (1) no polymer secretion and no quorum sensing (EPS^−^), (2) constitutive polymer secretion and no quorum sensing (EPS^+^), and (3) polymer secretion under negative quorum-sensing control such that EPS secretion stops at high cell density (QS^+^). A fourth strain for which polymer secretion is under positive quorum-sensing control is omitted from the main analysis because its behavior was found to be qualitatively identical to that of the EPS^+^ strain (see Discussion, Text S1, and Figure S1). Our simulations examine quorum-sensing control of a single trait (EPS) in response to the concentration of a single autoinducer. In reality, bacteria often use more than one autoinducer to regulate multiple traits, and indeed, several quorum-sensing circuits may be linked via parallel or serial signaling pathways within the cell [15,16,41]. There is a rich scope, therefore, for additional study of many potential complexities of quorum-sensing–regulated social behaviors, which we leave open here.

### Model Framework

Biofilm development involves a number of interacting physical and biological processes, including growth, neighbor-pushing, solute diffusion, and other cell–cell and cell–solute interactions, all of which occur largely at the spatial scale of single cells. We use individual-based modeling methods to explore the emergent characteristics of these processes at the level of whole biofilms [42]. Simulated cells behave independently according to user-defined kinetic rate expressions designed to represent the essential features of bacterial metabolism. Our simulations begin with one or more colonizing cells, which are attached to a uniformly flat surface and grow in a two-dimensional (2-D) space with horizontal periodic boundary conditions. The model framework used here allows the definition of any number and kind of bacterial and solute species [43]. As cells consume substrate according to their strain-specific metabolism kinetics and produce additional biomass, they grow and divide once a maximum cell radius is achieved. Movement of cells, which are modeled as rigid circles, results from forces exerted between neighbors as they grow and divide. Summed over all the cells present, these forces cause the biofilm front to advance. Solutes diffuse across a boundary layer between the biofilm and a bulk fluid in which solute concentrations are assumed to be homogeneous and constant. Inside this boundary layer, we determine the dynamics of solute spatial distributions by solving the 2-D diffusion-reaction equations. In so doing, we assume that solute concentrations reach their diffusion-reaction equilibria much faster than bacterial cells grow and divide [43,44]. The biofilm simulation framework and its associated numerical methods have previously been described in detail [42,43,45].

### Strain Definitions

Following Xavier and Foster [36], we assume that bacteria consume a substrate, S, and invest it in the production of biomass and EPS (for a full list of model notation, see Table 1). This allows a simple definition of the strains based upon their biomass versus EPS investment strategies. Non-EPS producers (EPS^−^) devote all substrate taken up to biomass production, whereas unconditional EPS producers (EPS^+^) always allocate a proportion *f* to EPS synthesis.

**Table 1:**
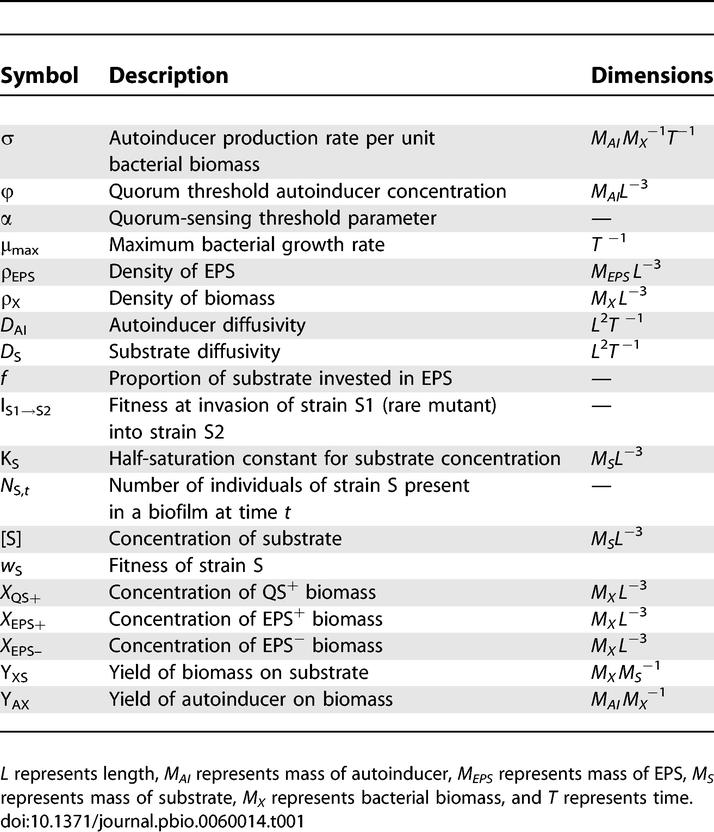
Notation Summary

Our third strain, QS^+^, is intended to represent a hypothetical first step in the evolution of quorum sensing. We assume that QS^+^ cells have gained the ability to detect a waste chemical produced by conspecific bacteria. This chemical can be envisioned as a byproduct of metabolism or cellular housekeeping that has been co-opted as a primitive autoinducer for monitoring local population density. This scenario is consistent with many real-world autoinducers, especially those of Gram-negative bacteria and some unicellular yeasts, which are closely related to, or simply are, metabolic waste products [4,15,46,47]. One way that the transition from a nonresponsive to a responsive quorum-sensing phenotype could occur is through mutation in a preexisting transcription factor, which allows it to bind the accumulating autoinducer. Binding the autoinducer may then alter the transcription factor's ability to control the expression of an EPS synthase. This abstraction conforms very well with the molecular mechanism underlying LuxI/R-type quorum-sensing circuits widely observed among bacteria [4,15,48].

Bacteria grow according to Monod saturation kinetics, and we assume that all cells secrete an autoinducer without cost and at a constant rate (Table 2). Following the pattern exhibited by V. cholerae, QS^+^ cells synthesize EPS only when local autoinducer concentration is below the quorum-sensing threshold concentration. Once this threshold level is exceeded, QS^+^ cells terminate EPS synthesis and invest only in biomass production [9]. The timing and density dependence with which QS^+^ bacteria reach a quorum depends upon three key factors: (1) how quickly the autoinducer is produced, (2) how quickly the autoinducer diffuses away from the biofilm, and (3) the critical quorum-sensing autoinducer concentration. For example, fast autoinducer production, slow autoinducer diffusion, and a low critical quorum-sensing autoinducer concentration will all lead to a quorum being reached more quickly and at lower cell density. To account for the dependence of quorum-sensing behavior on all of these factors, we group them into a single parameter, 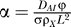 , where σ is the autoinducer production rate per unit bacterial biomass, *D_AI_* is the autoinducer diffusion coefficient, and ϕ is the quorum-sensing threshold autoinducer concentration. ρ_X_, the bacterial biomass density, and *L*, the length of the biofilm simulation space, are included in α to form a dimensionless group. Using a dimensionless group to describe the quorum-sensing process allows us to make qualitative predictions that are independent of the specific values of the parameters contained in α, albeit within the bounds of systems that have these physical properties.

**Table 2:**
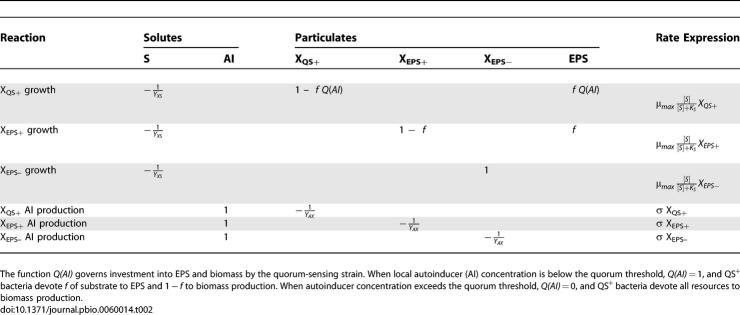
Stoichiometry of Bioprocesses Included in Simulations

Strains with the same α value will reach their respective quorums at the same time after the initiation of biofilm growth, irrespective of the different potential combinations of σ, *D_AI_*, ϕ, ρ_X_, and *L* that can produce a particular α value. Although α accounts for multiple factors that simultaneously contribute to quorum-sensing dynamics, to aid intuition, one may hold all parameters other than ϕ constant and think of α as the critical quorum-sensing autoinducer concentration. α simply measures how readily QS^+^ cells switch from low to high cell-density state: for higher α, QS^+^ bacteria will reach a quorum at higher cell density and relatively later on in the course of biofilm growth.

In order to determine whether simple quorum-sensing behavior (QS^+^) provides a fitness advantage over the unconditional behavioral strategies EPS^+^ and EPS^−^, we first consider competition in mixed biofilms initialized with the same number of either (1) QS^+^ and EPS^+^ or (2) QS^+^ and EPS^−^. We replicate these simulations over a range of α values for the QS^+^ strain in order to examine how the timing and density dependence of quorum sensing influence the outcome of competition.

### Simple Competition: QS^+^ versus EPS^+^, and QS^+^ versus EPS^−^

**Figure 1:**
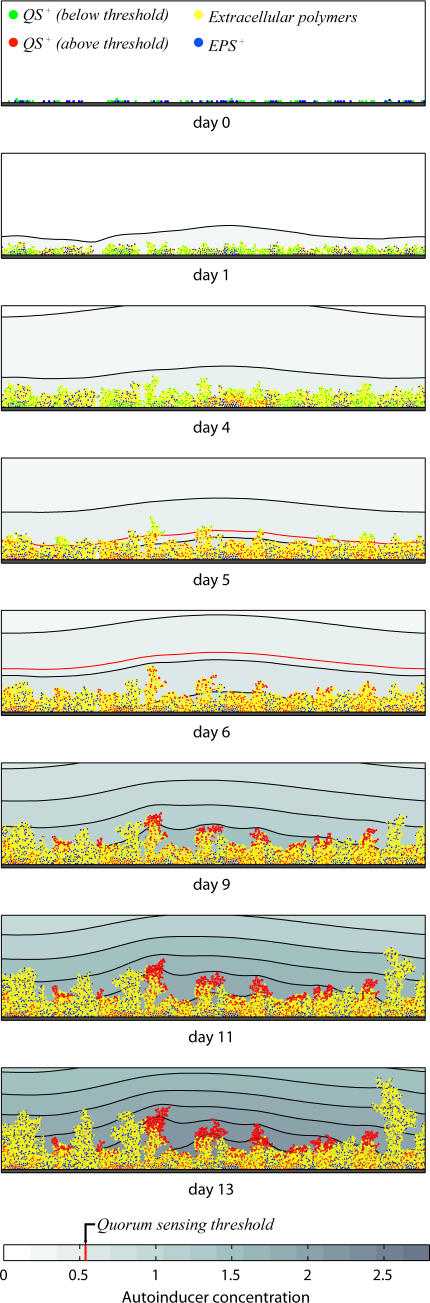
Direct Competition between QS^+^ and EPS^+^ Bacteria Initialized with Equal Numbers of Both Strains Autoinducer (AI) concentration is shown in the background, where isoconcentration lines represent 0.1-mg/l steps. Both strains behave identically, producing both EPS and biomass, until the autoinducer quorum-sensing threshold is reached. QS^+^ cells then turn off polymer secretion, devote all resources to biomass production, and achieve a growth burst at locations on the upper surface of the biofilm where substrate availability is highest. A movie for this simulation is provided as Video S1.

Simulations were parameterized with empirically estimated values (Table 3), initialized with 50 cells of each strain placed randomly on the solid substratum, and allowed to run for 14 simulated days (Figure 1), which is close to the maximum duration of a V. cholerae infection [49]. The proportion of energy invested in EPS secretion (*f*) will determine the extent to which EPS production allows one strain to displace others from a biofilm. As Xavier and Foster have discussed [36], for a given set of simulation parameters, there exists some evolutionarily stable strategy for EPS production, f*, which will out-compete any strain that invests either more or less in EPS. To find this optimum strategy, we performed an evolutionary stability analysis in which EPS^+^ strains with incrementally larger or smaller *f* values were competed against each other (see Text S1 and Figure S2). We found that, for our model conditions, the evolutionarily stable strategy for EPS investment independent of quorum sensing is approximately f* = 0.5, which was used for both the EPS^+^ and the QS^+^ strains (when below its quorum) in all subsequent simulations.

**Table 3:**
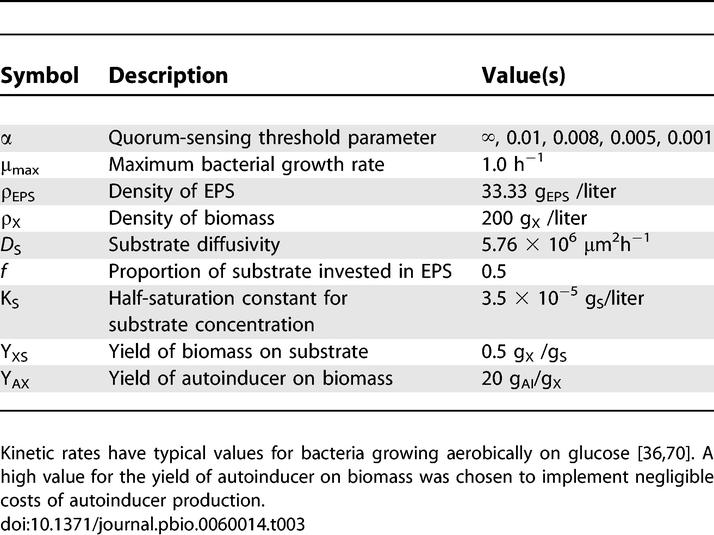
Parameters Used in Biofilm Simulations

**Figure 2:**
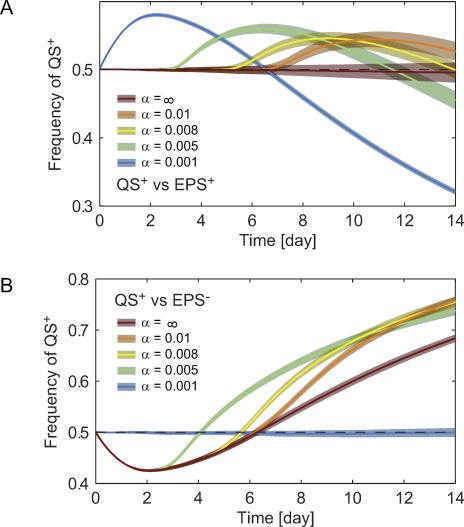
Summary of Simple Competitions (A) A quorum-sensing strain that down-regulates polymer secretion at high cell-density (QS^+^) is competed against a constitutive polymer-secreting strain (EPS^+^). (B) QS^+^ versus non-polymer producer (EPS^−^). Each competition (QS^+^ vs. EPS^+^, and QS^+^ vs. EPS^−^) was replicated 50 times for each of the α values: ∞, 0.01, 0.008, 0.005, and 0.001, where α captures how quickly the QS^+^ strain will switch from low to high cell-density state (see main text). For higher α, QS^+^ bacteria will reach their quorum at higher cell density, relatively later on during biofilm growth. Plotted lines represent mean QS^+^ frequency time series from each set of 50 simulations and are shown with shaded 95% confidence intervals. Note that in (A) and (B), the plotted lines corresponding to α = ∞ are control treatments in which QS^+^ behaves identically to EPS^+^ throughout simulations because autoinducer concentrations never reach the QS^+^ quorum-sensing threshold. Similarly, in (A) and (B), the plotted lines corresponding to α = 0.001 are control treatments in which QS^+^ behaves identically to EPS^−^ throughout simulations because autoinducer concentrations always exceed the QS^+^ quorum-sensing threshold.

Competitions between the QS^+^ and EPS^+^ strains and between the QS^+^ and EPS^−^ strains were repeated for a range of α values. We included two controls, one (α = ∞) in which the QS^+^ strain never reaches its quorum and behaves identically to the EPS^+^ strain, and another (α = 0.001) in which the QS^+^ strain reaches a quorum immediately after simulations begin, and behaves identically to the EPS^−^ strain thereafter. The frequency of QS^+^ cells within the biofilm was calculated at each time step and averaged over all replicate simulations to generate a mean QS^+^ frequency plot for each α value used in both sets of competitions (Figure 2A and 2B).

#### Competition between QS^+^ and EPS^+^.

In a mixed competition between the quorum-sensing strain and a constitutive EPS producer, all cells are initially phenotypically identical; that is, they all secrete EPS. However, as cells grow and population density increases, the autoinducer accumulates, and at a time point dependent upon their α value, quorum-sensing (QS^+^) cells turn off polymer secretion and invest all their resources in growth. Near the upper surface of the biofilm, where substrate availability is highest, QS^+^ cells achieve a burst of cell division (Figure 1, days 9–13). In the short term, the QS^+^ strain increases in frequency over and above that of the constitutive EPS producer. The advantage is temporary, however, because the EPS^+^ strain continues to secrete polymer and eventually produces towers that suffocate neighboring QS^+^ cells (see for example Figure 2A, α = 0.005), analogous to the case of competing EPS^+^ and EPS^−^ cells [36]. Quorum-sensing control of EPS production, therefore, provides a competitive advantage over constitutive EPS production, but only for a limited time window. Moreover, the location of this window within the period of biofilm growth is determined by how quickly the QS^+^ strain reaches a quorum. Strains with higher α attain growth bursts later in the course of biofilm formation (Figure 2A).

#### Competition between QS^+^ and EPS^−^.

Without having to pay the cost of EPS production, EPS^−^ cells rapidly divide at the beginning of simulations and achieve a higher initial frequency than QS^+^ cells. By secreting EPS, however, the QS^+^ strain rises up and over the top of neighboring cells, suffocating those that do not secrete polymer. After its initial disadvantage due to lower growth rate, the QS^+^ strain rapidly ascends to a majority in the biofilm and remains there indefinitely. Unlike the EPS^+^ strain, QS^+^ cells switch to pure biomass production after they have suffocated their EPS^−^ neighbors; at this point, investment into EPS is no longer advantageous. As a result, the QS^+^ strain will out-compete non-EPS producers by even larger margins than the constitutive EPS producer (Figure 2B).

### Rare-Mutant Invasion Analysis

The simple competition simulations described above suggest that bacteria for which EPS production is under quorum-sensing control have a time-dependent advantage over strains that are not capable of responding to changes in population density. However, a within-group competitive advantage need not translate into evolutionary success when the advantage comes at a strong cost to overall productivity [50]. More concretely, if successfully suppressing another strain in a biofilm causes the entire biofilm to grow poorly, the net effect on fitness may be deleterious [36]. We investigated this possibility through evolutionary invasion analyses to determine whether rare-mutant QS^+^ cells can increase in frequency in populations of either EPS^+^ or EPS^−^ cells, and whether a successful QS^+^ strain, once in the majority, can subsequently resist invasion by rare EPS^+^ and EPS^−^ mutants. To do this, we simply compare the number of cell divisions of the invading strain in a focal biofilm to the mean number of cell divisions by the majority strain taken across all biofilms in the population. More formally, we first define the fitness of a bacterial strain as the average number of cell divisions that it achieves on a defined time interval [0, *t_end_*]:

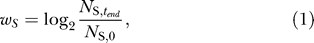
where *N*_S*,t*_ is the number of cells of strain S present within the biofilm at time *t*. Letting S1 be a rare mutant, we define its ability to invade a majority strain, S2, as follows:

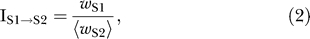
where *w*_S1_ is the fitness of the potential invader (S1) in direct competition with S2, as described in Equation 1, and 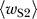 is the mean fitness of S2 cells in a pure S2 biofilm, which approximates mean fitness in the population. We assume that the bacterial population as a whole contains many more biofilms than the focal simulated biofilm in which the potential invading strain (S1) has arisen. All biofilms other than the focal simulated biofilm are composed purely of the resident strain, S2, and contribute vastly more to mean population fitness. Therefore, 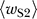 effectively measures the fitness of S2 cells when competing solely with other S2 cells. For a rare-mutant S1 to invade a majority strain S2, 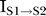 must be greater than unity; that is, S1 must fare better against S2 than S2 fares against itself [51].

*Length of biofilm tenure*: A key variable in this analysis is the time interval [0, *t_end_*] on which *w*_S1_ and 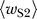 are measured. When choosing *t_end_*, we are asking: at what point during biofilm growth is it critical for long-term evolutionary success to be in the majority? We take the answer to be the time at which dispersal or disturbance occurs, and we assume that all cells within a biofilm have an equal probability of entering the propagule pool from which subsequent biofilms are seeded. This approach takes into consideration both local competition within biofilms and global competition between biofilms to determine the long-term evolutionary success of an invading bacterial strain [50]. Importantly, our method of analyzing invasiveness also assumes that dispersal or disturbance occurs in one large burst at a discrete point in time, rather than continuously throughout biofilm growth (see Discussion).

*Genetic relatedness at biofilm initiation*: We performed reciprocal invasion analyses using simulated competitions between QS^+^ and EPS^+^ or QS^+^ and EPS^−^ with a range of initial QS^+^ frequencies. This captures the effect of a rare mutant entering a population of another strategy, where the starting frequency of the rare strain reflects the number of strains randomly inoculated, and therefore the initial average relatedness, within the biofilm. For example, if 10 strains are present at the initiation of each biofilm, then a rare mutant will begin at a local frequency of 0.1, and average relatedness within the biofilm where the rare mutant resides will start at 0.1 [2,36].

#### Invasion analysis: QS^+^ and EPS^+^.

**Figure 3:**
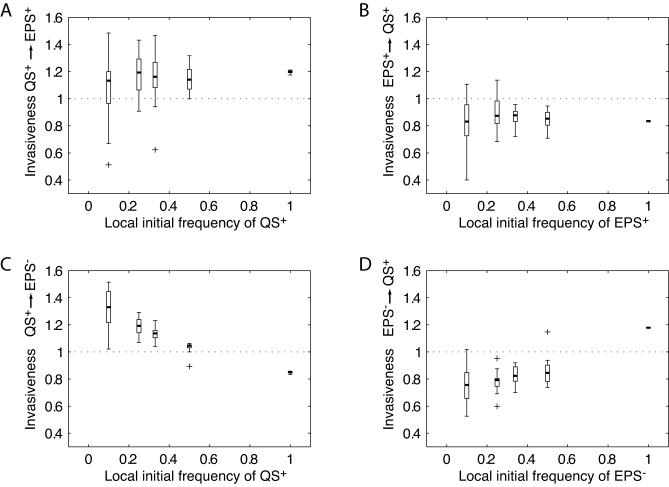
The Quorum-Sensing Strain Can Invade Non-Quorum-Sensing Strains, but Not Vice Versa Invasiveness of a rare mutant was analyzed for different degrees of mixing among strains in biofilms, reflected in the different initial frequencies of the rare strain in the biofilm. For example, if 10 strains are randomly sampled, then the initial frequency of the rare mutant in its own biofilm will be 0.1; initial relatedness will also be 0.1 (see main text). Each box and whisker plot summarizes the results of 20 replicate simulations, and plus signs (+) denote outliers. All simulations were carried out at α = 0.008 for the QS^+^ strain. (A) Invasion of a rare quorum-sensing strain (QS^+^) into a population of unconditional EPS producers (EPS^+^), and (B) failure of a rare EPS^+^ strain to invade a population of QS^+^ bacteria. Biofilms composed entirely of QS^+^ cells attain higher average fitness than biofilms composed entirely of EPS^+^ cells. (C) Invasion of a rare QS^+^ strain into a population of non-EPS producers (EPS^−^), and (D) failure of a rare EPS^−^ strain to invade a population of QS^+^ bacteria. Again, the QS^+^ strain can invade EPS^−^, whereas EPS^−^ cannot invade QS^+^. Notably, however, biofilms composed entirely of QS^+^ cells have a lower average fitness than biofilms composed entirely of EPS^−^ cells. Therefore, if all biofilms contained only a single genotype (no within-biofilm evolutionary competition), the EPS^−^ would invade and resist invasion.

We investigated whether a quorum-sensing strain that obtains an advantage in single biofilms (Figures 1 and 2) can invade a population of constitutive EPS producers and resist their reinvasion. We therefore focus on parameter values under which the QS^+^ strain has an advantage in the simple competition simulations. Specifically, we examine invasiveness for a disturbance interval of 9 d (*t_end_* = 9), with a QS^+^ strain α value (QS sensitivity) of 0.008, and we find that the QS^+^ strain can readily invade populations composed mostly of EPS^+^ cells, but not vice versa (Figure 3A and 3B). Additionally, biofilms composed entirely of QS^+^ cells have a higher average fitness than biofilms composed entirely of EPS^+^ cells.

#### Invasion analysis: QS^+^ and EPS.^−^

Again using *t_end_* = 9 d, we find that the QS^+^ strain invades a resident population of EPS^−^ cells, whereas the reverse is not true (Figure 3C and 3D). It is notable, however, that biofilms composed entirely of QS^+^ cells have a lower mean fitness than biofilms composed entirely of EPS^−^ cells, which reflects the fact that investment into EPS reduces total biomass production and therefore average growth rate.

## Discussion

Biofilm formation and quorum sensing are central and often interconnected features of bacterial social life [4–6,15,28,29]. Our evolutionary analysis is the first to address both of these major classes of bacterial social behavior, and it suggests that quorum sensing enables bacteria to turn on and off the secretion of extracellular polymeric substances (EPS) so as to increase their competitive ability against other species and strains within biofilms. This result builds upon the conclusions of Xavier and Foster [36], who predicted that EPS secretion affords an advantage to secreting strains in competition with nonsecreting strains. It is important to note that this view contrasts with the conventional wisdom that EPS is a public good that simply binds the biofilm together and protects it against external threats [52,53], although a combination of these perspectives is also a realistic possibility. We modeled both positive and negative quorum-sensing regulation of EPS production. Though explicit simulations of the planktonic phase were omitted, it seems clear that secreting EPS at high cell density allows cells to selectively activate EPS synthesis in biofilms and avoid the cost of EPS production in the planktonic phase [36]. However, we also find potential benefits for down-regulating EPS at high cell density, which allows cells to redirect energy from EPS production into growth and cell division prior to a dispersal event (Figure 1). Such a quorum-sensing phenotype will only be favored if detachment events are predictable, due to consistent extrinsic disturbance, or if dispersal is induced by the bacteria themselves.

Our findings are consistent with the known biology of V. cholerae, which exhibits negatively quorum-sensing–regulated EPS secretion. The environments that V. cholerae occupies appear to present opportunities for EPS-mediated competition within biofilms. Although we do not yet know how often different V. cholerae strains compete within human hosts, it is clear that infections always involve multiple species. These include other pathogenic genera, such as *Pseudomonas*, *Salmonella*, and *Campylobacter* [54,55], and there is compelling evidence that V. cholerae must compete with native intestinal microbial fauna in order to become established [56]. Furthermore, EPS secretion appears to be important for within-biofilm competition: quorum-sensing–deficient V. cholerae mutants that overproduce EPS take over biofilm cultures coinoculated with wild-type bacteria [9].

The ecology of pathogenic V. cholerae is characterized by cycles of rapid growth followed by massive dispersal events; the bacteria effect a stereotypical disease progression from initial infection, through the formation of biofilm-like aggregates [57], to release from the intestinal tract after enormous toxin-induced fluid release. This suggests that quorum sensing in V. cholera can be tuned to coincide with purging from the gut. Interestingly, quorum-sensing mutants that overproduce EPS suffer a greatly decreased ability to escape from biofilms [58–60], which indicates that, in addition to saving energy, reducing EPS secretion also actively assists dispersal. Moreover, on reaching a quorum, V. cholerae produces a protease whose putative function is to facilitate detachment [9,39,58,59]. By secreting a “detachase” and down-regulating EPS production at high cell density, V. cholerae appears to be inducing a growth burst coincident with efficient dispersal. The cycle of growth and detachment may also play a role in the initial colonization of the host: cells in a biofilm formed early in an infection can, upon detecting a threshold autoinducer concentration, halt EPS secretion, detach, and seed other areas of the intestine.

Whereas V. cholerae terminates EPS secretion at high cell density, many other species, including the opportunistic human pathogen P. aeruginosa, activate EPS secretion at high cell density. Hammer and Bassler [9] suggested that the explanation for this stark contrast in quorum-sensing behavior may lie in different infection strategies. Our results support this argument and, furthermore, suggest that this divergence hinges upon the evolutionary tradeoff between within-biofilm competition on the one hand and dispersal ability on the other. In particular, chronic infections are less likely to involve discrete and predictable moments of detachment that would favor a clear cutoff point for polymer secretion. Instead, dispersal is likely to occur through many small events over a long, but indeterminate, period of time. In such conditions, strains that can dominate locally, thereby maximizing their chances of detachment over an interval of uncertain length, will attain an evolutionary advantage. We therefore predict that up-regulation of EPS secretion at high cell density, which focuses resource investment into sustained local competitive ability, is more likely to be favored. This is precisely the pattern exhibited by P. aeruginosa, which is notorious for the chronic, and often terminal, infections it establishes in the lungs of cystic fibrosis patients. Interestingly, populations of P. aeruginosa sampled from the cystic fibrosis lung often also contain quorum-sensing mutants that are fixed in a high cell-density state [61] and a low cell-density state [33,62], although the link between these results and the EPS secretion phenotype, if any, is not yet clear.

The biofilm simulations performed in this study highlight several hypotheses amenable to experimental testing. We anticipate that EPS production by V. cholerae is at least partially a competitive behavior in the human intestinal tract, as it is in lab biofilm assays [9]. Although we lack a direct test of this prediction, Nielsen et al. [60] found that V. cholerae mutants unable to produce EPS are just as effective at colonizing rabbit intestine as wild-type cells, which shows that EPS is not secreted simply to aid surface colonization. The same study found that *rpoS*, which encodes an important stationary-phase regulator, is necessary for escape from the intestinal wall, implying that the detection of nutrient starvation also regulates dispersal [60].

A comparison of different V. cholerae strains offers additional opportunities to test our conclusions. Natural isolates show considerable variation in quorum-sensing ability, with strains fixed in either low or high cell-density states [63]. Our simulations raise the possibility that variation in quorum-sensing state within V. cholerae is linked to different dispersal requirements across the bacterium's diverse ecology. V. cholerae strains are known to form biofilms on both biotic and abiotic surfaces in marine environments [64–66], and not all cause disease [63]. Specifically, we predict that pathogenic strains selected for rapid colonization of, and efficient dispersal from, human hosts or other temporary environments will exhibit negatively quorum-sensing–regulated EPS production. The Classical V. cholerae biotype, which was responsible for the first six global cholera pandemics, has a nonfunctional copy of a key regulatory protein, HapR, involved in the quorum-sensing response. However, in line with our predictions, it was recently discovered that these strains are capable of HapR-independent quorum sensing and may still repress EPS expression in response to high cell density [67]. The associated prediction is that strains that occupy single locations for long periods should accumulate mutations that enable constitutive EPS production in biofilms, regardless of local population density. In support of this, standing cultures of EPS^−^ V. cholerae cells are reliably taken over by spontaneous, constitutive EPS^+^ mutants [9].

Cooperation, competition, and communication are all intertwined in microbial communities, and we are only beginning to unravel the processes that drive this rich interaction [1,2,68,69]. Although our simulations inevitably miss many biological details of any one species or strain, a familiar principle of sociobiology emerges. A full understanding of quorum sensing in bacterial biofilms will require consideration of evolutionary competition within and among these social groups.

## Supporting Information

Figure S1 Summary of Simple Competition Involving the QS* Strain, Which Up-Regulates Polymer Secretion at High Density (A) Competition between the QS* strain and the constitutive EPS-secreting strain (EPS^+^).
(B) Competition between the QS* strain and the non–EPS-secreting strain (EPS^−^). These simulations differ from those carried out for Figure 2 (main text); here, the QS* strain produces no EPS at low cell density and initiates EPS secretion only after autoinducer concentration exceeds the threshold value. Each competition was repeated 50 times, and plotted lines represent mean QS* frequency time series from each set of simulations, shown with shaded 95% confidence intervals.
(1.0 MB EPS)

Figure S2 An Evolutionary Stability Analysis for Investment into EPS (*f*) Each box-and-whisker plot summarizes the results of 20 replicate simulations.
(A) Invasion analysis (see Equation 1, main text) of EPS^+^ strains with slightly higher *f* values than the rest of the population (*f* − Δ*f*) yields f* = 0.52.
(B) Invasion analysis of EPS^+^ strains with slightly lower *f* values than the rest of the population (*f* + Δ*f*) yields f* = 0.45. Together, these two analyses demonstrate that the evolutionarily stable strategy for EPS investment, f*, lies between 0.45 and 0.52, and *f =* 0.5 was used for the simulations in our main text. The value of Δ*f* used for this evolutionary stability analysis was 0.1. Focal biofilms were initiated with an equal number of cells of each type (average relatedness of 0.5), and invasiveness was calculated using *t_end_* = 14 d (see main text).
(502 KB EPS)

Text S1 Simulation of a Bacterial Strain that Up-Regulates EPS Production (QS*) at High Cell Density in Competition with Constitutive EPS Producers (EPS^+^) and Non-Producers (EPS^−^), and an Evolutionary Stability Analysis for Investment into EPS Secretion (44 KB DOC)

Video S1 Movie File for the Simulation Shown in Figure 1
Also available for download at: http://sysbio.harvard.edu/csb/foster/joao/QSposVsEPSpos_alpha8e-3_seed1.mov.
(3.6 MB MOV)

## Abbreviations

EPS: extracellular polymeric substance
EPS^+^: constitutive extracellular polymeric substance-producing strain
EPS^−^: non-extracellular polymeric substance-producing strain
QS^+^: quorum-sensing strain
