# Supplementary material for: The Evolution of Quorum Sensing in Bacterial Biofilms
Source: PLoS Biol. 2008 Jan 29;6(1):e14. doi: 10.1371/journal.pbio.0060014 (PMC2214811; doi:10.1371/journal.pbio.0060014)
Supplement: Text S1 — (44 KB DOC) [file pbio.0060014.sd001.doc]

# The evolution of quorum sensing in bacterial biofilms

**Supporting Information**

Carey D. Nadell ^1*†^, Joao B. Xavier ^2†^, Simon A. Levin^1^, and Kevin R. Foster ^2*^

^1^ Department of Ecology and Evolutionary Biology, Princeton University, Princeton, NJ 08544

^2^ Center for Systems Biology, Harvard University, Bauer Laboratory, 7 Divinity Avenue, Cambridge, MA 02138

^*^ Authors for correspondence. CDN: [cnadell@princeton.edu](mailto:cnadell@princeton.edu); KRF: [kfoster@cgr.harvard.edu](mailto:kfoster@cgr.harvard.edu)

† These authors contributed equally to this work.

**Contents**

1. Analysis of positive quorum-sensing regulation of EPS production
2. Evolutionary Stable Strategy (ESS) for polymer investment

**1. Analysis of a strain with positively-quorum-sensing-regulated EPS production**

The main text focuses upon three strains:

1. EPS^–^ : No polymer secretion and no quorum sensing
2. EPS^+^ : Constitutive polymer secretion and no quorum sensing
3. QS^+^ : Negatively-quorum-sensing-regulated polymer secretion.

The fourth possibility is a strain for which polymer secretion falls under positive quorum-sensing control. This strain, here named QS*, is omitted from the main text because the three strains above are sufficient to capture the essential dynamics. Here, we analyze the competitive dynamics of strain QS* against other strains and demonstrate that the behavior of this strain in biofilms is well described by EPS^+^.

When growing in a well-mixed planktonic phase, a bacterial population lacks the spatial structure of a biofilm. As a result, polymer production cannot aid in competing for superior spatial locations and only carries a metabolic cost, leading to decreased growth rate. Positively-quorum-sensing-regulated polymer excretion clearly can be advantageous, since polymer production in the planktonic phase is simply a waste of resources. QS* strains that are capable of waiting until a high cell density is reached (as in a biofilm) before initiating EPS production can therefore enjoy a selective advantage.

When in a biofilm, however, the QS* strain behaves like EPS^–^ until the autoinducer concentration accumulates to the threshold level, after which the QS* strain behaves like EPS^+^. As described in the main text for the QS^+^ strain, the parameter α sets the timing with which the QS* strain switches from low-cell density state (EPS^–^ behavior) to high cell density state (EPS^+^ behavior). The higher the value of α, the longer the QS* strain will wait before initiating EPS secretion. The value of α is therefore important for the outcome of competition between the QS* strain and the EPS^+^ or EPS^–^ strain. It is particularly noteworthy that the optimal α is either the highest possible, which would make QS* behave exactly like EPS^–^, or the lowest possible, which would make it behave exactly like EPS^+^, depending upon the length of the typical biofilm lifespan.

To illustrate this point, we have performed simple competition simulations. When QS* competes against EPS^+^ (Figure S1A) we see that a QS* strain that waits any length of time before initiating EPS secretion enjoys an advantage against EPS^+^ only when biofilm lifetimes are short. In these conditions, the advantage is maximized when α is so large that the QS* strain never reaches a quorum and never produces EPS (i.e., behaves identically to EPS^–^). Conversely, when biofilm lifetimes are long, the QS* strain should begin secreting EPS as soon as possible. In other words, α will evolve to be so low that the QS* strain reaches its quorum and begins to secrete EPS at the lowest possible cell density that typically characterizes the biofilm state. Hence, the outcome of competition between the QS* strain and both the EPS^+^ and EPS^-^ strains is the following: short-term competitions favor high α (EPS^–^ behavior) and long-term competitions favor low α (EPS^+^ behavior).

Most importantly, the activation of EPS secretion at high cell density is observed in strains typically associated with long biofilm lifetimes, such as *P. aeruginosa*, which causes chronic infections in the lungs of cystic fibrosis patients. When biofilm lifetimes are long, QS* strains will be selected for minimal α, which leads to EPS^+^ behavior. Thus, for the conditions relevant to our current study, a quorum-sensing strain that up-regulates EPS secretion at high cell density is qualitatively identical to an EPS^+^ strain that always produces EPS in the biofilm.

**2. Evolutionary Stable Strategy (ESS) for investment into extracellular polymer production**

We carried out an evolutionary stability analysis to determine the optimal investment into extracellular polymeric substances (EPS). Xavier and Foster [1] demonstrated that EPS secretion provides a competitive advantage in biofilms for the strains producing it, despite the energetic cost that it carries. This occurs because bacterial lineages of EPS producers are propelled more efficiently towards nutrient-rich areas in a way that smothers competitors. This study also demonstrated the existence of an optimal investment into EPS, defined as an evolutionarily stable strategy. This optimal value is expected to vary with biological and environmental parameters and hence must be determined again for the particular parameter set considered here (Table 3, main text).

We considered the evolution of EPS investment strategies (proportion of resource uptake devoted to EPS, denoted *f*) through competition of EPS^+^ strains with mutations of small effect. We first determined the invasiveness of strains with a given *f* value (the invading strain) into populations of strains with slightly less polymer production (*f* –Δ*f*). This revealed that it is advantageous to invest more into EPS, but only up to *f =* 0.52, at which point invasiveness equals unity (Figure S2A). Beyond this value, as discussed previously [1], there is no longer a competitive advantage of excreting more polymer because too much EPS spreads cells in the biofilm so that nutrient gradients become very shallow, eliminating the advantage of being pushed toward higher spatial locations.

We then determined the invasiveness of strains with a given *f* value (the invading strain) into populations of strains with slightly higher polymer secretion (*f +*Δ*f*). This analysis indicated that strains producing less EPS can invade high EPS producers down to values of *f* = 0.45 (Figure S2B).

Together, these two analyses demonstrate that an evolutionarily stable level of investment into EPS, *f**, exists between 0.45 and 0.52. We use *f* = 0.5 for the EPS^+^ and QS^+^ strains in the primary simulations for this study.

**References**

1. Xavier JB, Foster KR (2007) Cooperation and conflict in microbial biofilms. Proc Natl Acad Sci U S A 104: 876-881.
